# Drastic improvement of cardiac function after living-donor renal transplantation in a long-term hemodialysis patient with severe mitral regurgitation

**DOI:** 10.1186/s40981-022-00583-8

**Published:** 2022-12-09

**Authors:** Satoko Noguchi, Junichi Saito, Eiji Hashiba, Kazuyoshi Hirota

**Affiliations:** Department of Anesthesiology, Hirosaki University Graduate School of Medicine, 5 Zaifu-Cho, Hirosaki, Aomori, 036-8562 Japan

**Keywords:** Renal transplantation, Long-term hemodialysis, Mitral regurgitation

## Abstract

**Background:**

Although there are reports of recovery of cardiac function after renal transplantation, the feasibility of renal transplantation in patients with low cardiac function remains controversial.

**Case presentation:**

A 59-year-old Japanese male was scheduled to undergo living-donor renal transplantation (LDRT) under general anesthesia. Preoperative transthoracic echocardiography revealed severe mitral regurgitation (MR) and a left ventricular ejection fraction (LVEF) at 30%. LDRT was conducted prior to cardiac surgery with restrictive fluid management and close monitoring of cardiac function. The patient’s renal function improved promptly after the LDRT, and his hemodynamics were stable throughout the perioperative period. Along with improvements in the patient’s renal function and anemia, the patient’s cardiac function improved to LVEF 50% and achieved drastically improved MR as well as cardiac function, without intervention.

**Conclusion:**

This case indicates that LDRT has the potential to improve cardiac function in patients who have been on hemodialysis for more than 20 years.

## Background

Renal transplantation for patients with end-stage renal disease (ESRD) has a high cardiovascular risk during the perioperative period. Avoiding renal transplantation has been the standard strategy for the proper use of grafts, especially for patients with severely impaired cardiac function [1]. However, evidence that renal transplantation can improve these patients’ cardiac function has been accumulating [2]. The indications for living-donor renal transplantation (LDRT) for patients with cardiac impairment remain controversial. In particular, it is unclear whether LDRT can improve low cardiac function status in patients on long-term dialysis. We report the case details of a long-term dialysis patient with severe mitral regurgitation (MR) and a low left ventricular ejection fraction (LVEF) whose cardiac function improved after he underwent LDRT.

The patient’s written consent to publish this case report was obtained.

## Case description

A 59-year-old Japanese male (height 171 cm, weight 69.2 kg) was scheduled to undergo LDRT from his brother under general anesthesia. He had been treated for chronic kidney disease due to polycystic kidney disease, and he had begun hemodialysis 21 years ago. He had also been taking medications for atrial fibrillation (Af) and MR for 9 years, and he underwent a percutaneous coronary intervention to the left anterior descending artery for angina pectoris 5 years ago. There had been no history of heart failure symptoms or associated hospitalization since then.

The blood test revealed decreased kidney function with a serum creatinine of 10.49 mg/dL and blood urea nitrogen of 51 mg/dL, and mild anemia with hemoglobin of 10.0 g/dL. The patient’s brain natriuretic peptide value was 971 pg/mL. Transthoracic echocardiography before the LDRT showed severe MR and a left ventricular ejection fraction (LVEF) at 30%, with low cardiac output. Coronary computed-tomography angiography showed no stent stenosis but three-vessel disease was suspected, which led to the conclusion that cardiac dysfunction was due to ischemic cardiomyopathy. Restrictive pulmonary dysfunction with vital capacity of 2.7 L (65% of predictive value) was also observed. The priority of surgery for the patient’s severe MR or for his ESRD was discussed, and it was concluded that further preoperative assessment was required to determine the indications for surgery.

Transthoracic echocardiography and right heart catheterization were then conducted to evaluate the impacts of a preload reduction induced by arteriovenous shunt obstruction and of a dobutamine infusion on the patient’s cardiac output (CO) and pulmonary artery pressure. The preload reduction resulted in no changes in pulmonary hypertension or the severity of the patient’s MR, and a continuous intravenous dobutamine infusion increased his CO and pulmonary artery pressure (Table 1). The patient’s mitral valve had no mechanical abnormality, and the MR was secondary due to cardiac ischemia. Based on these findings, we decided to perform LDRT before cardiac surgery with restrictive fluid management and a close monitoring of the patient’s cardiac function.

**Table 1:** Preoperative cardiac function

		Before obstruction of AV shunt	After obstruction of AV shunt	After CIV DOB 5 μg/kg/min	After CIV DOB 10 μg/kg/min
PAC	CO, L/min	4.3	4.2	4.9	5.8
	CI, L/min/m^2^	2.3	2.2	2.6	3.1
	PCWP, mmHg	18	19	17	21
	PAP, mmHg	40/19/29	42/17/30	55/16/32	58/22/35
TTE	SV, mL	53	50	57	61
	MR	3+	3+	3+	3+

*AV* arteriovenous, *CI* cardiac index, *CIV* continuous intravenous, *CO* cardiac output, *DOB* dobutamine administration, *MR* mitral regurgitation, *PAC* pulmonary artery catheter, *PAP* pulmonary artery pressure, *PCWP* pulmonary capillary wedge pressure, *SV* stroke volume, *TTE* transthoracic echography

Oral midazolam 10 mg and roxatidine acetate 75 mg were administered as an anesthetic premedication 1 h before the LDRT surgery. In addition to the standard ASA monitoring, the patient’s invasive artery pressure, pulmonary artery pressure, and CO were measured. Anesthesia was induced and maintained with propofol, ketamine, and fentanyl. After an administration of rocuronium 50 mg, the trachea was intubated without any complications. The end-tidal carbon dioxide was maintained at 35–40 mmHg. A continuous infusion of dobutamine 1 μg/kg/min effectively increased cardiac index from 2.6 to 3.4 L/min/m^2^ and maintained the urine outflow after graft anastomosis. The intraoperative data were as follows: 1570 mL crystalloid fluid administration, 450 mL colloid fluid administration, 327 g estimated blood loss, and 810 mL urine output; duration of surgery, 225 min; duration of anesthesia, 304 min. After the patient’s tracheal tube was displaced in the operating room, he was transferred to the intensive care unit (ICU) for the observation of cardiac and renal function. At 3 days post-LDRT, the serum creatinine level had decreased to 1.27 mg/dL, and although the patient gained 2 kg, his hemodynamics were stable without complaints.

**Fig. 1:**
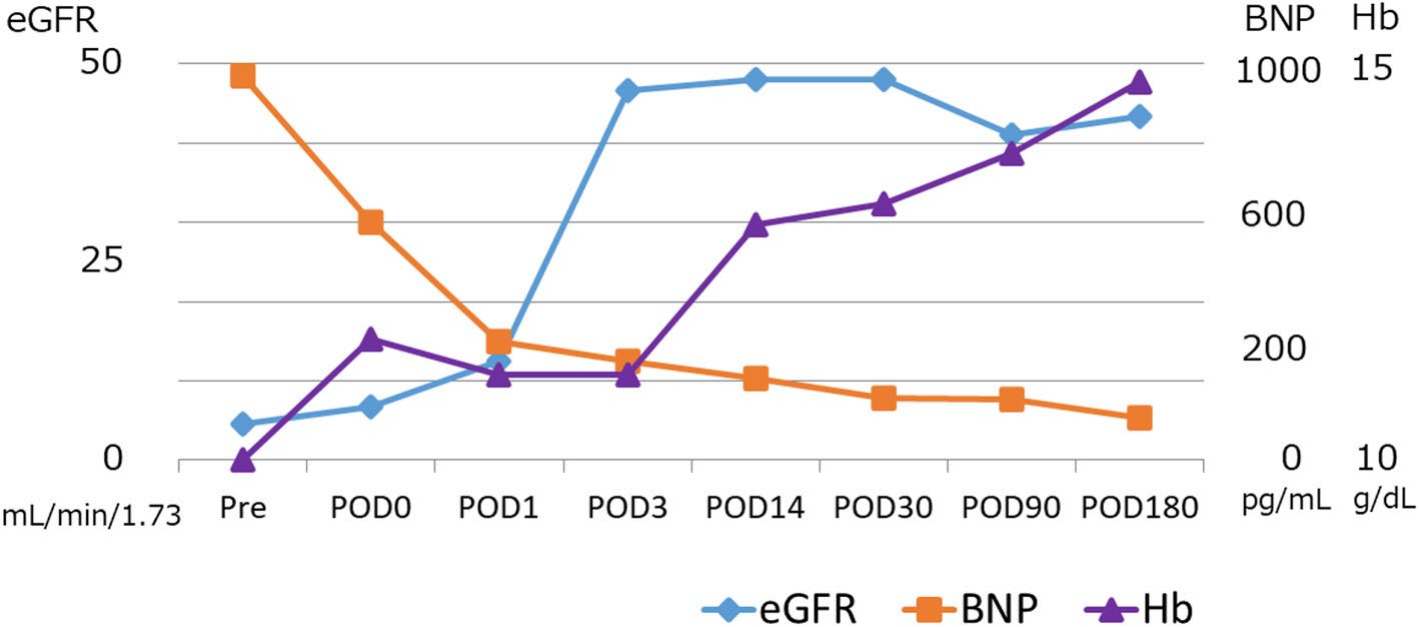
Changes in the perioperative cardiac and renal function; the patients’ cardiac and renal function, and anemia improved greatly. BNP, brain natriuretic peptide; eGFR, estimated glomerular filtration rate; Hb, hemoglobin; POD, postoperative day

**Fig. 2:**
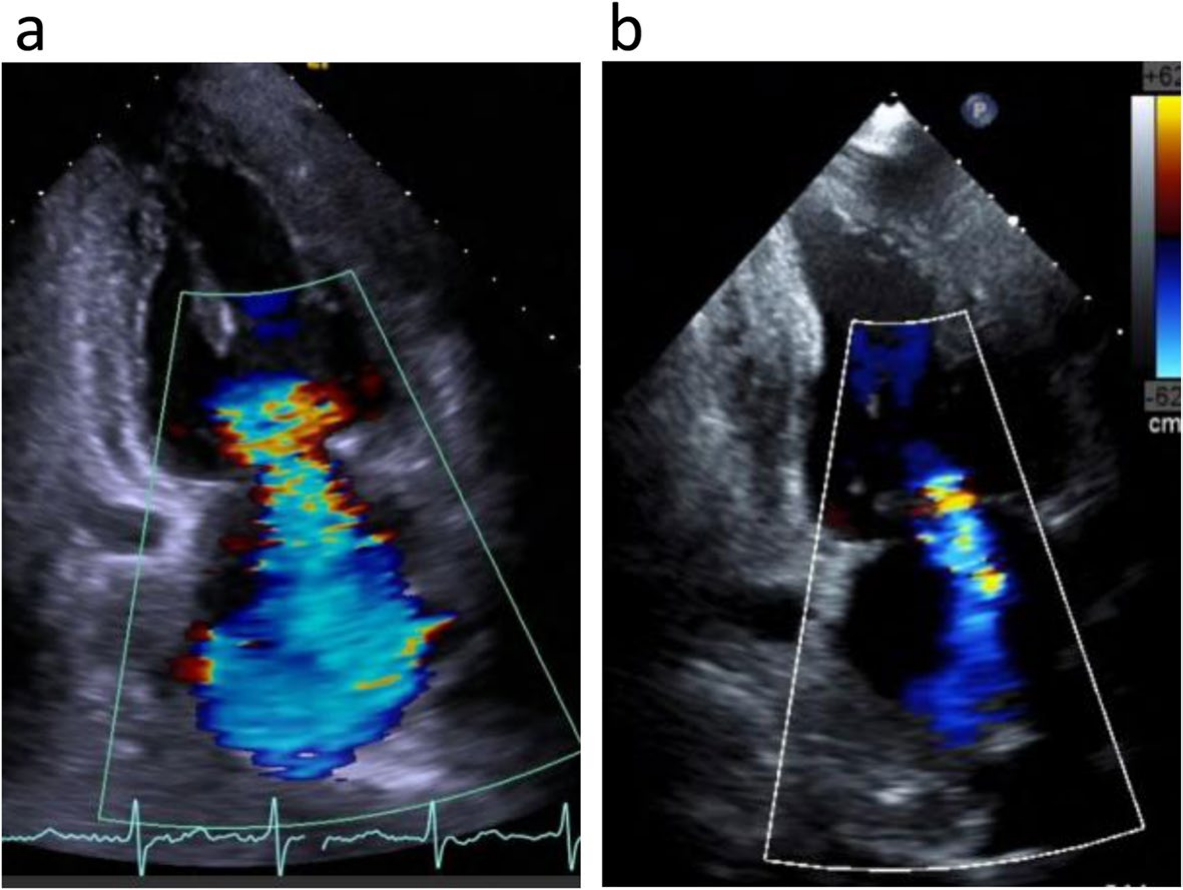
Changes in cardiac function before (**a**) and after (**b**) living-donor renal transplantation by transthoracic echocardiography. Left ventricular ejection fraction, left ventricular end-diastolic diameter, and left ventricular end-systolic diameter were 30%, 57 mm, and 50 mm at the preoperative point (**a**) and 50%, 50 mm, and 38 mm at postoperative day 180 (**b**), respectively. Severity of mitral regurgitation, effective regurgitant orifice area (proximal isovelocity surface area), and tricuspid regurgitant pressure gradient were severe, 0.25 cm^2^, and 30 mmHg at the preoperative point (**a**) and mild, 0.12 cm^2^, and 21 mmHg at the postoperative day 180 (**b**), respectively. The above results show that the patient’s cardiac function and severe mitral regurgitation improved to a degree that indicated that cardiac surgery was no longer necessary

During the postoperative course, the patient’s cardiac and renal function and anemia were dramatically improved (Fig. 1). As his cardiac function remained stable after the LDRT, cardiac surgery for his MR has been unnecessary (Fig. 2).

## Discussion

The priority of valvular surgery to non-cardiac surgery has been indicated by several guidelines; however, the influence of end stage renal disease on it has not been considered in those guidelines, and priority of valve surgery to renal transplantation is not known [3]. Normal structure of the mitral valve leaflets is often maintained in patients with functional MR secondary to decreased left ventricular function as observed in our case [4]. Renal transplantation may thus precede cardiac surgery in patients in whom improvement in cardiac function is expected after renal transplantation. However, the perioperative outcome data are not yet sufficient to justify a prior renal transplantation in patients with asymptomatic severe valvular lesions, and further studies are needed to accumulate such data.

The key finding in the present case is that even in a patient who had been on dialysis for > 20 years, improved renal function after an LDRT was accompanied by improved cardiac function, and unnecessary intervention to the heart was avoided. Several reports have indicated that renal transplantation recovered cardiac function in patients who were younger with shorter duration of dialysis than the present case [5, 6]. Renal transplantation also improved cardiac function due to severe MR in a patient with diabetic nephropathy before introducing hemodialysis [7]. According to Wali et al., approx. 70% of renal transplant recipients with an LVEF ≤ 40% showed an improvement of their LVEF to ≥ 50% at 2 months after renal transplantation [8]. They also revealed that long-term dialysis was a factor preventing normalization of the LVEF. Based on our experience with the present patient, we speculate that long-term hemodialysis does not prohibit an improvement in cardiac function after renal transplantation.

Renal failure and cardiac disease are closely related [9]. Mild anemia, probably due to renal failure existed before surgery. Because anemia itself increases the ischemia, oxidative stress, and inflammation [10], it may have worsened the cardiac function of this patient. Cardiac function was improved after renal transplantation as shown by the decrease of BNP. Recovery of renal function improved anemia through an increase of erythropoietin secretion, which may have contributed to the improvement in the cardiac function.

Perioperative management after renal transplantation often involves aggressive fluid loading to promote diuresis in order to avoid intravascular dehydration. However, in our patient’s case, fluid loading and dilution caused anemia and excessive preload, which are likely to easily cause heart failure due to severe MR. A pulmonary artery catheter was therefore inserted in order to monitor changes in the patient’s CO, while minimal IV fluids and catecholamines were administered.

This case report demonstrates that even in a patient who has been on dialysis for more than 20 years, the improved renal function after LDRT can improve the patient’s cardiac function and avoid unnecessary intervention to the heart. The present patient with severe MR and impaired cardiac function was able to undergo surgery and obtained significant benefit after LDRT. Renal transplantation should thus be considered as a treatment for ESRD patients with decreased cardiac function.

## Data Availability

Please contact author for data requests.

## Abbreviations

ESRD: End-stage renal disease
ICU: Intensive care unit
LDRT: Living-donor renal transplantation
LVEF: Left ventricular ejection fraction
MR: Mitral regurgitation
